# Genetic parameters and genome-wide association analysis of service sire effects on litter size and its relationship with boar semen quality in three terminal sire lines

**DOI:** 10.1186/s12711-026-01071-8

**Published:** 2026-07-30

**Authors:** Ching-Yi Chen, Daniela Lourenco, Michael Kleve-Feld, Adria S. Bhatnagar, Justin W. Holl

**Affiliations:** 1 The Pig Improvement Company, Genus plc, Hendersonville, TN USA; 2 Department of Animal and Dairy Science, University of Georgia, Athens, GA USA

## Abstract

**Background:**

Reproductive performance is key to efficient and profitable pig production. In selecting boars to enhance overall male reproductive success, the paternal genetic influence on litter size is of particular interest, in addition to semen quality improvement in terminal sire lines. Few studies have investigated the impact of service sire effects on litter size in swine. Previous models often treated the service sire as a non-genetic effect or as a genetic effect but ignoring the additive relationship between service sires and sows. Moreover, no studies have incorporated genomic information when examining service sire genetic effects for litter size in pigs. In this study, we aimed to estimate genetic parameters for service sire additive genetic effects on number born alive (NBA) and conducted a genome-wide association study (GWAS) using large genotyped data sets in pigs. Both service sire and sow effects were modeled as additive genetic effects, considering a covariance structure among them, given by the relationship matrix. The relationship between service sire additive genetic effects on NBA and direct additive genetic effects for boar semen quality traits was also evaluated.

**Results:**

Estimates of heritability ranged from 0.01 to 0.03 for service sire effects and from 0.09 to 0.15 for sow effects, with their genetic correlation estimates ranging from − 0.20 to 0.37. Results from GWAS showed no strong signals for service sire effects on NBA. Heritability estimates ranged from 0.10 to 0.12 for sperm motility and from 0.17 to 0.28 for total abnormal morphology. Correlations between service sire genomic estimated breeding values for NBA and semen quality were low but in the favorable direction.

**Conclusions:**

Selection for semen motility might be favorable for improving semen morphology. Although the service sire genetic contribution to litter size was small, the low but favorable correlations between genomic estimated breeding values for litter size and semen quality suggest that incorporating service sire effects together with semen quality traits into selection can improve boar reproductive performance.

## Background

Reproductive performance in terminal sire lines is typically focused on boar fertility traits, such as semen quality, whereas traits like litter size are primarily addressed through maternal line breeding objectives. However, to improve overall male reproductive success and better capture a terminal boar’s contribution to downstream reproductive outcomes, the paternal genetic influence on litter size is of particular interest, especially in artificial insemination systems, where a single sire can impact a large number of litters. Although litter size is largely determined by maternal components, paternal contributions from service sires can also influence it through biological and genetic aspects [1–3]. Cieleń and Sell-Kubiak [3] provided a comprehensive review of the importance and variability of paternal effects on sow reproductive traits. Biologically, male reproduction can have an impact on fertilization success through sexual maturity and sperm quality. Factors such as age at first mating or semen collection, sperm quality and quantity, boar libido, and testes characteristics can influence boar reproductive ability. For example, boars younger than six months are not yet fully developed, and their use for insemination is generally not recommended due to lower fertilization efficiency. Genetically the service sire can also affect litter size indirectly through its influence on embryo development and survival. Recognizing and integrating these paternal contributions into breeding programs can enhance reproductive efficiency and support the overall success of pig breeding programs.

Previous studies have investigated service sire effects on litter size in swine, either by modeling it as a random non-genetic effect uncorrelated with sow additive genetic effect [4–7], or by modeling it as an additive genetic effect correlated with sow additive genetic effect [8–10]. With availability of computer-assisted sperm analysis (CASA) technology [11], artificial insemination centers can now routinely assess boar semen quality through automated estimates of various parameters, including sperm motility and morphology.

Literature on genetic parameters of semen quality traits reveals low to moderate heritability [6, 12–14] and a wide range of genetic correlations with sow litter size traits [6, 13, 15]. Although some studies have examined the genetic influence of the service sire on litter size by modeling it as an additive genetic effect correlated with sow additive genetic effect, reports incorporating genomic information in such analyses remain limited. These include applications of single-step genomic best linear unbiased prediction (ssGBLUP) [16] and single-step approach genome-wide association study (ssGWAS) in large genotyped swine populations.

The ssGWAS was developed by Wang et al. [17] to estimate marker effects from an ssGBLUP analysis. To measure statistical significance for resulting marker effect estimates, Aguilar et al. [18] expanded the method proposed by Lu et al. [19] to derive p-values for single-marker GWAS within the ssGWAS framework. Aguilar et al. [18] subsequently validated the algorithm using a large beef cattle dataset. All p-values were obtained in a single run with negligible computational time. Misztal et al. [20] indicated that there was a soft limit of about 100 K genotyped animals when obtaining p-values by inverting the left-hand side of the mixed model equations. An alternative approach that bypasses the need to invert the left-hand side components was developed by Bermann et al. [21], thereby eliminating the bottleneck in approximating reliabilities for large-scale genotyped populations. In their study, they extended the work from Misztal and Wiggans [22] on approximating prediction error (co)variances of GEBVs for the algorithm for proven and young (APY) core set and validated the approach using a beef cattle data set with about 330 K genotyped animals. Leite et al. [23] applied the approximation of p-value for ssGWAS on 450 K genotyped beef cattle.

In this study, we aimed to evaluate the paternal influence on litter size and its GEBV correlations with semen quality traits in terminal sire lines and to identify genomic regions associated with service sire effects on litter size using ssGWAS. To our knowledge, our study is the first to investigate service sire genetic effects on litter size in pigs using a large genotyped dataset.

## Methods

### Data

Data were provided by PIC (a Genus company, Hendersonville TN, USA). For litter size, the data set contained 6416, 23,188, and 48,890 farrowing records collected between 2020 and 2024, including from 3071, 11,819, and 24,089 sows mated to 197, 554, and 891 boars from terminal sire lines A, B, and C, respectively. This corresponded to an average of two litter records per sow and 33, 42, and 55 litters per service sire in lines A, B, and C, respectively. For semen quality, the data set contained 57,500, 608,996, and 346,059 ejaculates collected between 2013 and 2025 from 1698, 29,095, and 18,726 boars from terminal sire lines A, B, and C, respectively.

Semen quality traits of motility and total abnormal morphology were measured using the CASA system. Data quality controls were applied to exclude ejaculates with motility < 50% and abnormal morphology > 40%. These thresholds were selected based on the distribution of the study data to exclude low quality semen samples while retaining the majority of observations. Total abnormal morphology was calculated as the sum of tail miscellaneous abnormalities, head abnormalities, distal cytoplasmic droplets, and proximal cytoplasmic droplets.

The pedigree included 1,424,858, 4,344,630, and 2,146,583 animals for lines A, B, and C, respectively, among which 67,990, 259,250, and 365,392 animals were genotyped and imputed up to 50 K SNPs. Numbers of animals used in the analyses and the descriptive statistics for the 3 lines are shown on Table 1. The average number of piglets born alive ranged from 8.57 to 9.38. Average sperm motility ranged from 89.2 to 90.9%, while the average total abnormal morphology rate ranged from 9.5 to 12.2%.

**Table 1:** Descriptive statistics for each trait by line

Line	*N*	Mean	SD	Min	Max
*Number born alive*					
A	6,416	8.57	3.24	0	18
B	23,188	9.04	2.83	0	20
C	48,890	9.38	3.00	0	22
*Motility (%)*					
A	57,500	89.97	6.43	50	100
B	608,996	90.91	5.56	50	100
C	346,059	89.22	6.51	50	100
*Abnormal morphology (%)*					
A	57,500	11.25	6.51	0	40
B	608,996	9.52	6.20	0	40
C	346,059	12.17	8.06	0	40

For Number Born Alive = 0, records were retained in the analyses because they represent valid biological outcomes corresponding to complete stillbirths rather than recording errors

### Models and statistical analyses

Genetic parameters were estimated using BLUPF90 + with AIREML option [24].

#### Litter size genetic parameters

Analyses of NBA were performed using the following single-trait model:

1 $$\:\mathbf{y}=\mathbf{X}\boldsymbol{\upbeta\:}+{\mathbf{Z}}_{1}{\mathbf{u}}_{1}+{\mathbf{Z}}_{2}{\mathbf{u}}_{2}+{\mathbf{W}}_{1}{\mathbf{p}\mathbf{e}}_{1}+{\mathbf{W}}_{2}{\mathbf{p}\mathbf{e}}_{2}+\mathbf{e},$$

where $\:\mathbf{y}$ is the vector of phenotypes for NBA; $\:\boldsymbol{\upbeta\:}$ is the vector of fixed effects of parity group and contemporary group, defined as farm and farrowing year and month; $\:{\mathbf{u}}_{1}$ and $\:{\mathbf{u}}_{2}$ are the vectors of random additive service sire and sow genetic effects; $\:{\mathbf{p}\mathbf{e}}_{1}$ and $\:{\mathbf{p}\mathbf{e}}_{2}$ are the vectors of random service sire and sow permanent environmental effects; $\:\mathbf{e}$ is the vector of random residual effects; **X**, **Z**_1_, **Z**_2_, **W**_1_, and **W**_2_ are the incidence matrices relating records in **y** to effects in $\:\boldsymbol{\upbeta\:}$, $\:{\mathbf{u}}_{1}$, $\:{\mathbf{u}}_{2}$, $\:{\mathbf{p}\mathbf{e}}_{1}$, and $\:{\mathbf{p}\mathbf{e}}_{2}$ respectively. Random effects were assumed to follow multivariate normal distributions with means of zero and covariance structure as outlined below:

2 $$\:\mathrm{V}\mathrm{a}\mathrm{r}\left[\mathbf{u}\right]=\mathrm{V}\mathrm{a}\mathrm{r}\left[\begin{array}{c}{\mathbf{u}}_{1}\\\:{\mathbf{u}}_{2}\end{array}\right]=\:\mathbf{A}\otimes\:\left[\begin{array}{cc}{{\upsigma\:}}_{\mathrm{u}1}^{2}&\:{{\upsigma\:}}_{\mathrm{u}2}\\\:{{\upsigma\:}}_{\mathrm{u}12}&\:{{\upsigma\:}}_{\mathrm{u}2}^{2}\end{array}\right],$$

3 $$\:\mathrm{V}\mathrm{a}\mathrm{r}\left[\mathbf{p}\mathbf{e}\right]=\mathrm{V}\mathrm{a}\mathrm{r}\left[\begin{array}{c}{\mathbf{p}\mathbf{e}}_{1}\\\:{\mathbf{p}\mathbf{e}}_{2}\end{array}\right]=\:\mathbf{I}\otimes\:\left[\begin{array}{cc}{{\upsigma\:}}_{\mathrm{p}\mathrm{e}1}^{2}&\:0\\\:0&\:{{\upsigma\:}}_{\mathrm{p}\mathrm{e}2}^{2}\end{array}\right],$$

4 $$\:\mathrm{V}\mathrm{a}\mathrm{r}\left[\mathbf{e}\right]=\:\mathbf{I}\otimes\:{{\upsigma\:}}_{\mathrm{e}}^{2}$$

where **A** is the pedigree-based numerator relationship matrix; $\:\mathbf{I}$ is the identity matrix; $\:{{\upsigma\:}}_{\mathrm{u}1}^{2}$ and $\:{{\upsigma\:}}_{\mathrm{u}2}^{2}$ are the additive genetic variances of service sire and sow, with $\:{{\upsigma\:}}_{\mathrm{u}12}$ as their genetic covariance; $\:{{\upsigma\:}}_{\mathrm{p}\mathrm{e}1}^{2}\:$ and $\:{{\upsigma\:}}_{\mathrm{p}\mathrm{e}2}^{2}$ are the permanent environmental variances of service sire and sow; $\:{{\upsigma\:}}_{\mathrm{e}}^{2}$ is the residual variance. The service sire additive genetic effect represents the heritable component of paternal influence, while the service sire permanent environmental effect captures non-genetic, sire-specific influences that are consistent across repeated matings of the same sire. Temporary environmental effects associated with semen handling were assumed to be captured by the residual term rather than the service sire permanent environmental effect.

#### Semen quality genetic parameters

Analyses of semen quality traits were performed using the following two-trait model:

5 $$\:\mathbf{y}=\mathbf{X}\boldsymbol{\upbeta\:}+\mathbf{Z}\mathbf{u}+\mathbf{W}\mathbf{p}\mathbf{e}+\mathbf{e},$$

where $\:\mathbf{y}$ is the vector of phenotypes for MOT and ABN_MOR; $\:\boldsymbol{\upbeta\:}$ is the vector of fixed effects of contemporary group defined as farm and year and month at collection and the linear covariates of boar age at collection and rest days since the boar’s previous collection; $\:\mathbf{u}$ is the vector of random additive genetic effects; $\:\mathbf{p}\mathbf{e}$ is the vector of permanent environmental effects; $\:\mathbf{e}$ is the vector of random residual effects; **X**, **Z**, and **W** are the incidence matrices relating semen records in **y** to effects in $\:\boldsymbol{\upbeta\:}$, $\:\mathbf{u}$, and $\:\mathbf{p}\mathbf{e}$, respectively. Random effects were assumed to follow multivariate normal distributions with means of zero and covariance structure as outlined below:

6 $$\:\mathrm{V}\mathrm{a}\mathrm{r}\left[\mathbf{u}\right]=\mathrm{V}\mathrm{a}\mathrm{r}\left[\begin{array}{c}{\mathbf{u}}_{1}\\\:{\mathbf{u}}_{2}\end{array}\right]=\:\mathbf{A}\otimes\:\left[\begin{array}{cc}{{\upsigma\:}}_{\mathrm{u}1}^{2}&\:{{\upsigma\:}}_{\mathrm{u}12}\\\:{{\upsigma\:}}_{\mathrm{u}12}&\:{{\upsigma\:}}_{\mathrm{u}2}^{2}\end{array}\right],$$

7 $$\:\mathrm{V}\mathrm{a}\mathrm{r}\left[\mathbf{p}\mathbf{e}\right]=\mathrm{V}\mathrm{a}\mathrm{r}\left[\begin{array}{c}{\mathbf{p}\mathbf{e}}_{1}\\\:{\mathbf{p}\mathbf{e}}_{2}\end{array}\right]=\:\mathbf{I}\otimes\:\left[\begin{array}{cc}{{\upsigma\:}}_{\mathrm{p}\mathrm{e}1}^{2}&\:{{\upsigma\:}}_{\mathrm{p}\mathrm{e}12}\\\:{{\upsigma\:}}_{\mathrm{p}\mathrm{e}12}&\:{{\upsigma\:}}_{\mathrm{p}\mathrm{e}2}^{2}\end{array}\right],$$

8 $$\:\mathrm{V}\mathrm{a}\mathrm{r}\left[\mathbf{e}\right]=\:\mathbf{I}\otimes\:\left[\begin{array}{cc}{{\upsigma\:}}_{\mathrm{e}1}^{2}&\:{{\upsigma\:}}_{\mathrm{e}12}\\\:{{\upsigma\:}}_{\mathrm{e}12}&\:{{\upsigma\:}}_{\mathrm{e}2}^{2}\end{array}\right]$$

where **A** is the pedigree-based numerator relationship matrix; $\:\mathbf{I}$ is the identity matrix; $\:{{\upsigma\:}}_{\mathrm{u}1}^{2}$ and $\:{{\upsigma\:}}_{\mathrm{u}2}^{2}$ are the additive genetic variances for MOT and ABN_MOR, with $\:{{\upsigma\:}}_{\mathrm{u}12}$ as their genetic covariance; $\:{{\upsigma\:}}_{\mathrm{p}\mathrm{e}1}^{2}\:$ and $\:{{\upsigma\:}}_{\mathrm{p}\mathrm{e}2}^{2}$ are the permanent environmental variances, with covariance $\:{{\upsigma\:}}_{\mathrm{p}\mathrm{e}12}$; $\:{{\upsigma\:}}_{\mathrm{e}1}^{2}\:$ and $\:{{\upsigma\:}}_{\mathrm{e}2}^{2}$ are the residual variances, with covariance $\:{{\upsigma\:}}_{\mathrm{e}12}$.

#### Breeding value estimation

GEBVs were generated using ssGBLUP, which combines the pedigree-based relationship matrix, **A**, and the genomic relationship matrix (**G**) into a unified relationship matrix (**H**). Genomic relationship matrices compatible with pedigree information were investigated and developed previously [16, 25–29]. The $\:{\mathbf{H}}^{-1}$ was derived following Aguilar et al. [16] and Christensen and Lund [30] as:

9 $$\:{\mathbf{H}}^{-1}={\mathbf{A}}^{-1}+\left[\begin{array}{cc}0&\:0\\\:0&\:{\mathbf{G}}^{-1}-{\mathbf{A}}_{22}^{-1}\end{array}\right],$$

where $\:{\mathbf{A}}^{-1}$ is the inverse of the pedigree relationship matrix, $\:{\mathbf{A}}_{22}^{-1}$ is the inverse of the pedigree relationship matrix for genotyped animals, and $\:{\mathbf{G}}^{-1}$ is the inverse of the genomic relationship matrix. As reviewed by Lourenco et al. [24], implementation of APY in ssGBLUP enables highly efficient GEBV computation for large numbers of genotyped animals by constructing a sparse representation of $\:{\mathbf{G}}^{-1}$ and using an efficient algorithm to compute $\:{\mathbf{A}}_{22}^{-1}$ [31–33].

Pocrnic et al. [34] proposed that the number of core animals for APY could be defined according to the dimensionality of the genomic information represented by the genomic relationship matrix, which can be estimated as the number of eigenvalues explaining 98% of the variation in $\:\mathbf{G}$. For pigs, the optimal number of APY core animals was reported by Pocrnic et al. [34] to be approximately 6000.

In this study, the number of service sires across the three lines ranged from 197 to 891. For these datasets, relying solely on service sires would produce a core set that is too small. Therefore, core animals for each line included all service sires for that line, along with a set of randomly selected animals, for a core size of 20,000 animals per line.

#### Genome-wide association study

With the large number of genotyped animals being used in the study, we conducted ssGWAS using the method proposed by Leite et al. [23] to obtain the marker effects and approximate marker effect p-values. By combining the APY approach with an approximation of the prediction error (co)variance matrix for the GEBVs of the core animals, the computational burden was substantially reduced, enabling the estimation of marker effect p-values in large genotyped populations. The resulting p-values were adjusted for multiple testing using the Bonferroni correction. The genome-wide significance threshold was set at the 5% level, corresponding to -log10 (0.05/m), where m is the number of markers used in the respective analysis [18, 23].

## Results

### Genetic parameters

Estimates of variance components, heritabilities, and genetic correlations for NBA are presented in Table 2. For line A, the full model including permanent environmental effects for both service sire and sow failed to converge. Convergence issues remained when the model retained the service sire’s permanent environmental effect but excluded that of the sow. In contrast, convergence was achieved when the service sire’s permanent environmental effect was excluded and only the sow’s was retained. Therefore, a reduced model that excluded the service sire’s permanent environmental effect was used for line A, while the full model was applied for lines B and C. Model fit was evaluated using Akaike Information Criterion (AIC) by comparing models with and without the service sire permanent environmental effect. The AIC was substantially lower for the model with the service sire permanent environmental effect (49,784.19 and 131963.27 for lines B and C, respectively) compared with the model without this effect (80,874.43 and 186898.33 for lines B and C, respectively), supporting the inclusion of service sire permanent environmental effect in the model for lines B and C. Estimates of genetic variances attributed to the service sire were relatively small, accounting for approximately 27, 7, and 19% of the sow genetic variance estimates for lines A, B, and C, respectively. The estimate of the genetic covariance between service sire and sow was negative for line A but with high SE (-0.11 ± 0.13). Positive genetic covariance estimates were observed for lines B and C, with small SE (0.11 ± 0.03 and 0.08 ± 0.04). Estimates of permanent environment variances for service sire were less than 10% of those observed for the sow for lines B and C. Heritability estimates for the service sire were 0.03, 0.01, and 0.02 for lines A, B, and C, respectively. Corresponding heritability estimates for the sow were 0.11, 0.15, and 0.09. Estimates of genetic correlations between service sire and sow effects were − 0.20, 0.37, and 0.23 for lines A, B, and C, respectively. Estimates of repeatability for service sire effects were near zero for all lines, while repeatability estimates for sow effects were ranged from 0.18 to 0.24.

**Table 2:** Estimates of genetic parameters (SE) for Number Born Alive

	A	B	C
$\:{\sigma\:}_{u1}^{2}\:$	0.28 (0.08)	0.08 (0.03)	0.15 (0.04)
$\:{\sigma\:}_{u12}$	-0.11 (0.13)	0.11 (0.04)	0.08 (0.04)
$\:{\sigma\:}_{u2}^{2}\:$	1.04 (0.22)	1.16 (0.11)	0.79 (0.07)
$\:{\sigma\:}_{pe1}^{2}$	NA	0.01 (0.02)	0.06 (0.04)
$\:{\sigma\:}_{pe2}^{2}$	0.81 (0.19)	0.76 (0.09)	0.83 (0.07)
$\:{\sigma\:}_{e}^{2}$	7.32 (0.18)	5.95 (0.08)	6.92 (0.06)
$\:{h}_{1}^{2}\:$	0.03 (0.008)	0.01 (0.004)	0.02 (0.005)
$\:{h}_{2}^{2}\:$	0.11 (0.022)	0.15 (0.013)	0.09 (0.007)
$\:{rep}_{1}$	NA	0.01 (0.002)	0.02 (0.003)
$\:{rep}_{2}$	0.20 (0.018)	0.24 (0.010)	0.18 (0.006)
$\:{r}_{g}$	-0.20 (0.260)	0.37 (0.218)	0.23 (0.112)

$\:{\sigma\:}_{u1}^{2}\:,\:{{\sigma\:}_{u2}^{2},\:\sigma\:}_{pe1}^{2},{\:\sigma\:}_{pe2}^{2},\:{\sigma\:}_{e}^{2}$: service sire additive genetic, sow additive genetic, service sire permanent environment, sow permanent environment, and residual variances; $\:{h}_{1}^{2},\:{h}_{2}^{2}$: heritability for service sire and sow; $\:{rep}_{1},\:{rep}_{2}$: repeatability for service sire and sow; $\:{\sigma\:}_{u12}$, r_g_: genetic covariance and correlation between service sire and sow. Standard errors in parentheses

Estimates of variance components, heritability, and genetic correlations for MOT and ABN_MOR are presented in Table 3. For MOT, estimates of genetic variances were similar across the three lines (3.24 to 4.17), with a larger phenotypic variance of 42.9 for line A and smaller phenotypic variances of 26.8 and 31.4 for lines B and C. For ABN_MOR, estimates of genetic variances were nearly twice as large for line C (14.13) compared to other two lines (7.8 and 8.1), with similar phenotypic variances for lines A and C (45.3 and 51.0) and smallest phenotypic variances of 32.9 for line B. Overall, line B had the smaller estimates of non-genetic (permanent environmental and residual) variances among the three lines. Heritability estimates for MOT were 0.10, 0.12, and 0.11 for lines A, B, and C, respectively. For ABN_MOR, corresponding heritability estimates were 0.17, 0.25, and 0.28. Estimates of the genetic correlation (SE) between MOT and ABN_MOR were − 0.34 (0.21), − 0.39 (0.04), and − 0.64 (0.03) for lines A, B, and C, respectively. The corresponding phenotypic correlations were − 0.29 (0.001), − 0.30 (0.003), and − 0.38 (0.001) for lines A, B, and C, respectively. Estimates of repeatability ranged from 0.33 to 0.43 for MOT and from 0.50 to 0.54 for ABN_MOR. Correlations (SE) of GEBV between NBA and MOT were 0.13 (0.07), 0.06 (0.04), and 0.10 (0.03) for lines A, B, and C service sires, respectively. Corresponding estimates between NBA and ABN_MOR were − 0.12 (0.07), -0.08 (0.04), and − 0.12 (0.03) (Table 4).

**Table 3:** Estimates of genetic parameters (SE) for Motility and Abnormal Morphology

	A	B	C
$\:{\sigma\:}_{u1}^{2}\:$	4.17 (1.42)	3.24 (0.22)	3.33 (0.26)
$\:{\sigma\:}_{u12}$	-1.95 (1.24)	-1.99 (0.23)	-4.41 (0.38)
$\:{\sigma\:}_{u2}^{2}\:$	7.76 (1.90)	8.08 (0.40)	14.13 (0.80)
$\:{\sigma\:}_{pe1}^{2}$	14.31 (1.18)	7.75 (0.16)	7.13 (0.20)
$\:{\sigma\:}_{pe12}$	-8.60 (1.00)	-4.70 (0.16)	-5.20 (0.26)
$\:{\sigma\:}_{pe2}^{2}$	14.69 (1.42)	9.01 (0.26)	13.42 (0.52)
$\:{\sigma\:}_{e1}^{2}$	24.45 (0.15)	15.80 (0.03)	20.93 (0.05)
$\:{\sigma\:}_{e12}$	-6.74 (0.10)	-4.64 (0.02)	-6.06 (0.04)
$\:{\sigma\:}_{e2}^{2}$	22.85 (0.14)	15.85 (0.03)	23.46 (0.06)
$\:{h}_{1}^{2}\:$	0.10 (0.032)	0.12 (0.008)	0.11 (0.008)
$\:{h}_{2}^{2}\:$	0.17 (0.040)	0.25 (0.011)	0.28 (0.014)
$\:{rep}_{1}$	0.43 (0.011)	0.41 (0.003)	0.33 (0.003)
$\:{rep}_{2}$	0.50 (0.012)	0.52 (0.003)	0.54 (0.004)
$\:{r}_{g}$	-0.34 (0.209)	-0.39 (0.035)	-0.64 (0.030)

$\:{\sigma\:}_{u1}^{2}\:,\:{\sigma\:}_{u2}^{2}$: additive genetic variances for Motility and Abnormal Morphology; $\:{\sigma\:}_{pe1}^{2},{\:\sigma\:}_{pe2}^{2}:\:$permanent environment variances; $\:\:{\sigma\:}_{e1}^{2}\:,\:{\sigma\:}_{e2}^{2}:$ residual variances; $\:{h}_{1}^{2},\:{h}_{2}^{2}$: heritability; $\:{rep}_{1},\:{rep}_{2}$: repeatability; $\:{\sigma\:}_{u12}$, $\:{\sigma\:}_{pe12}$, $\:{\sigma\:}_{e12}$: genetic covariance, permanent environmental covariance, and residual covariances between Motility and Abnormal Morphology; r_g_: genetic correlation between Motility and Abnormal Morphology. Standard errors in parentheses

**Table 4:** Correlations (SE) between genomic estimated breeding values for the service sire effect on NBA and boar semen quality traits

Line	*N* of boars	Motility	Abnormal Morphology
A	197	0.13 (0.07)	-0.12 (0.07)
B	554	0.06 (0.04)	-0.08 (0.04)
C	891	0.10 (0.03)	-0.12 (0.03)

### Genome-wide association study

**Fig. 1:**
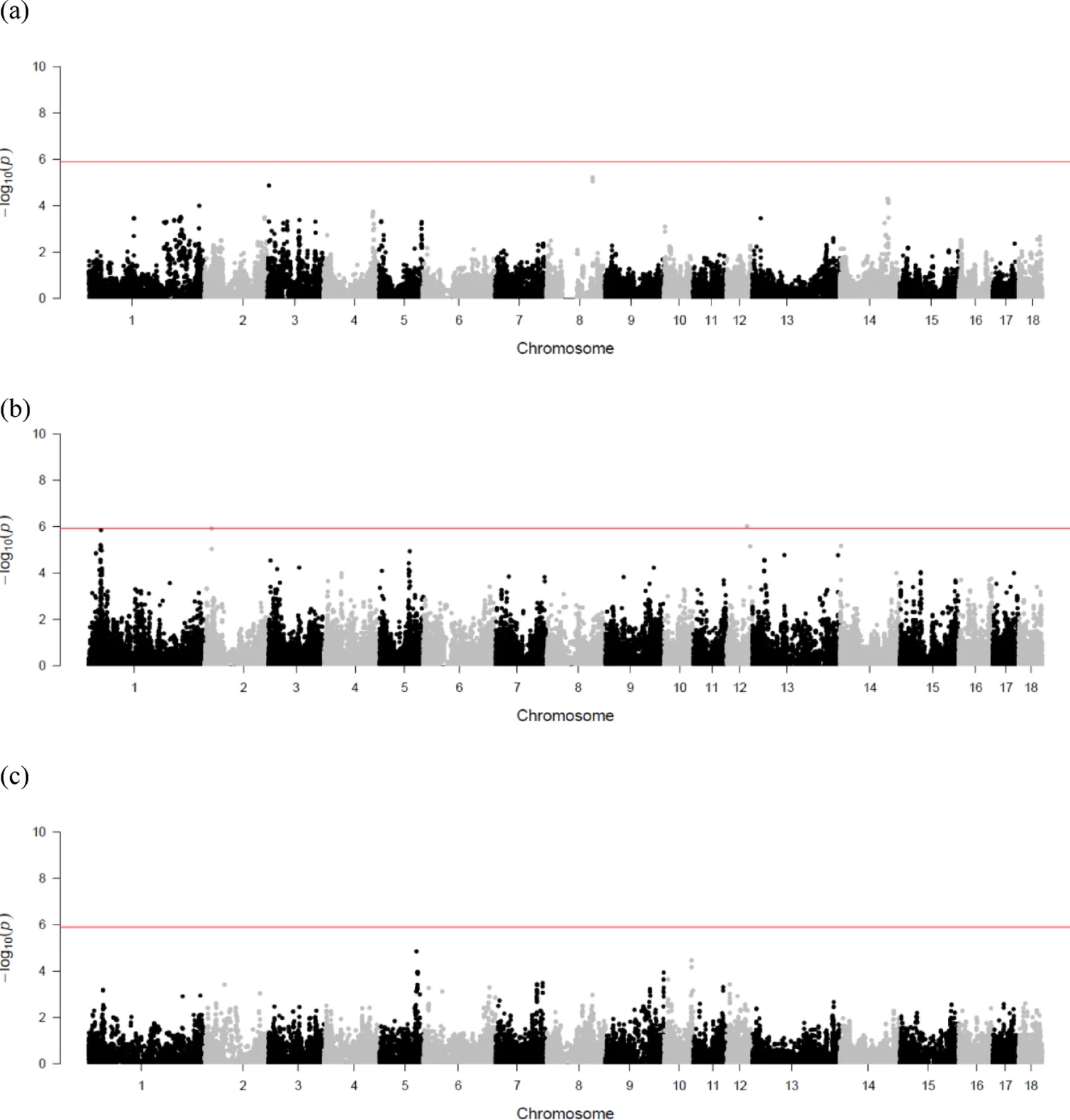
Manhattan plots of the single-step genome-wide association study for the genetic effect of service sire on Number Born Alive for **a** Line A **b** Line B **c** Line C. The x-axis represents chromosome numbers with the genomic coordinates of SNPs along the axis. The y-axis represents the observed -log10 P-value. The red line indicates the Bonferroni-corrected significance threshold at a nominal α = 0.05, which was − 5.90, -5.94, and − 5.88 for lines A, B, and C, respectively

**Fig. 2:**
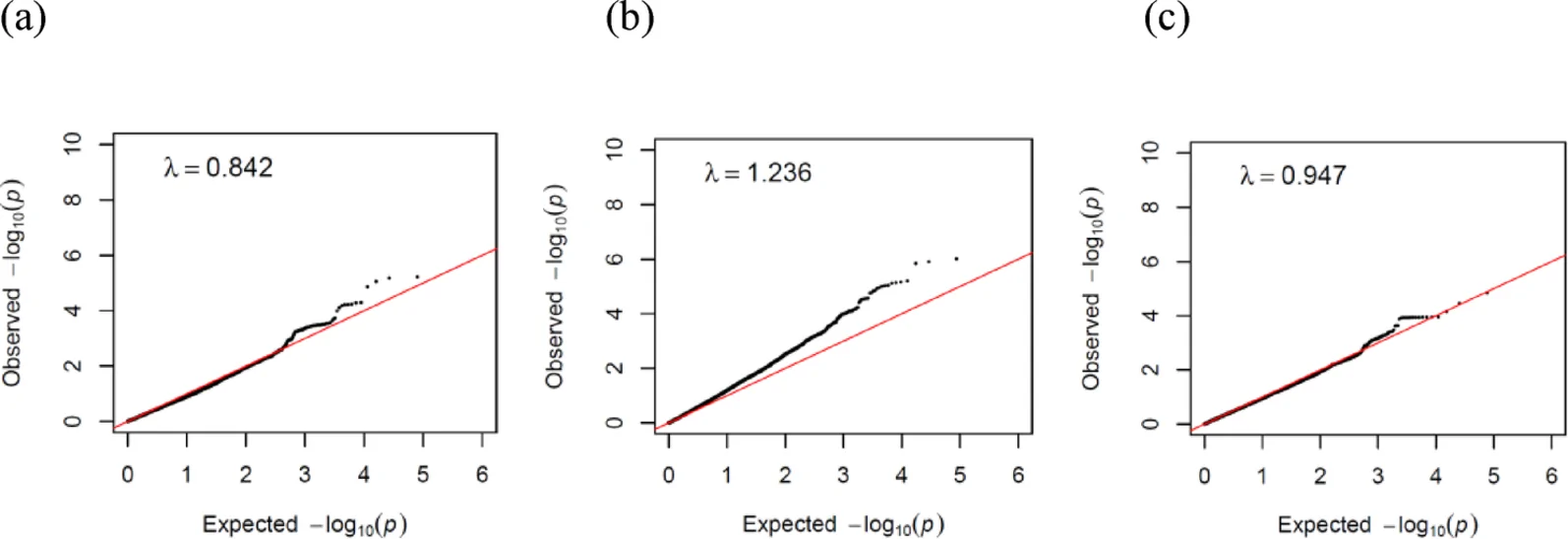
Quantile–quantile plots of the genome-wide association studies for genetic effect of service sire on Number Born Alive for **a** Line A **b** Line B **c** Line C

The -log10 of the Bonferroni-corrected rejection threshold for multiple testing at a nominal α = 0.05 was 5.90, 5.94, and 5.88 for lines A, B, and C, respectively. These thresholds were calculated as -log10(0.05/number of SNPs), based on 39,951, 43,505, and 38,228 SNPs for lines A, B, and C, respectively. Manhattan plots of -log10(P-value) for the genetic effect of service sire on NBA for the three lines are shown in Fig. 1. No significant peaks were identified for lines A and C. A marker on chromosome 12 near 48.29 Mb had -log10(P-Value) = 6.0 for the service sire genetic effect for line B. Quantile–quantile plots of the GWAS for the genetic effect of service sire on NBA are in Fig. 2. The genomic inflation factor (λ) of the QQ plot regression were 0.84, 1.24, and 0.95 for lines A, B, and C, respectively, indicating slight deflation for line A and slight inflation for line B.

**Fig. 3:**
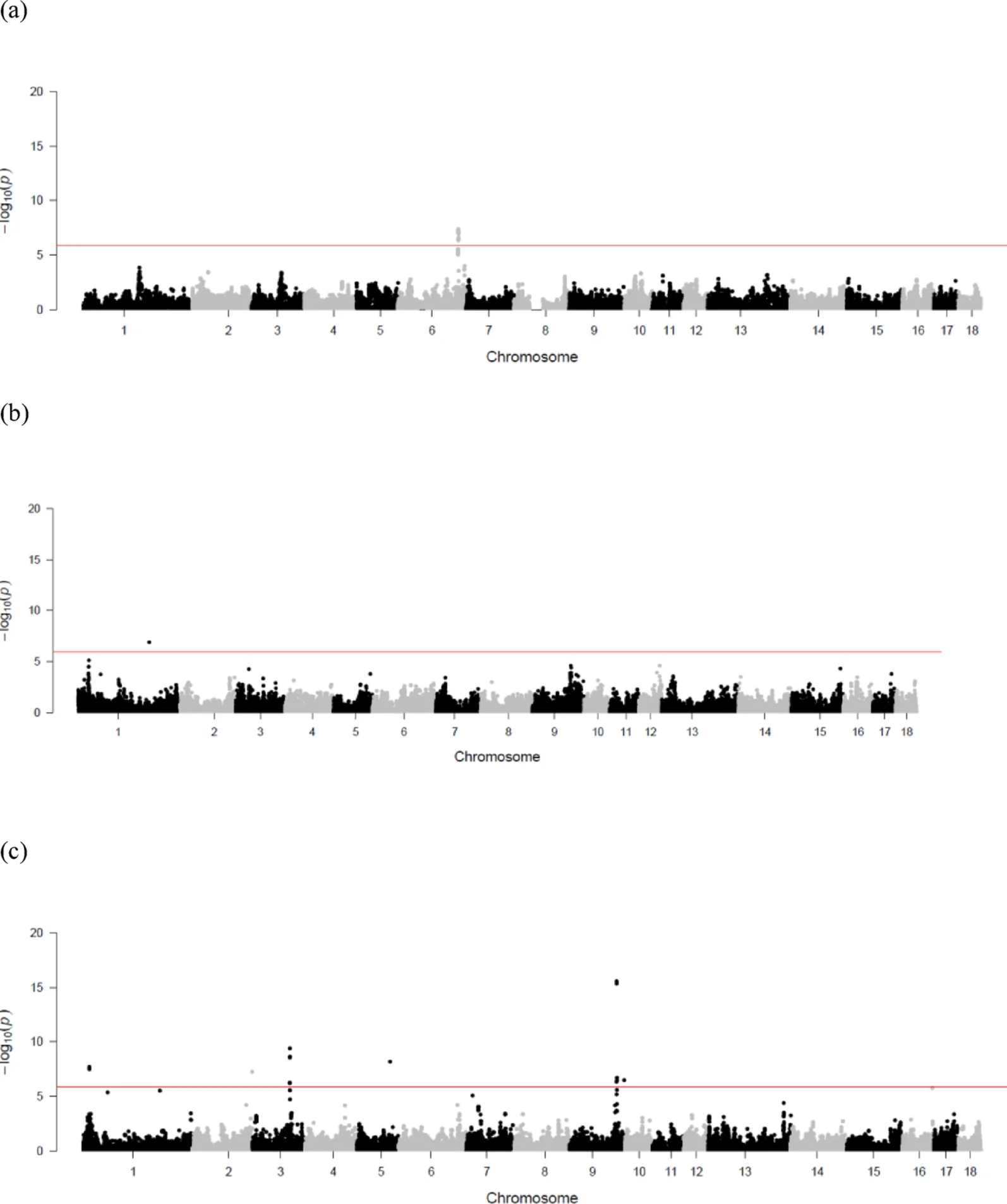
Manhattan plots of the single-step genome-wide association study for genetic effect of sow on Number Born Alive for **a** Line A **b** Line B **c** Line C. The x-axis represents chromosome numbers with the genomic coordinates of SNPs along the axis. The y-axis represents the observed -log10 P-value. The red line indicates the Bonferroni-corrected significance threshold at a nominal α = 0.05, which was − 5.90, -5.94, and − 5.88 for lines A, B, and C, respectively

**Fig. 4:**
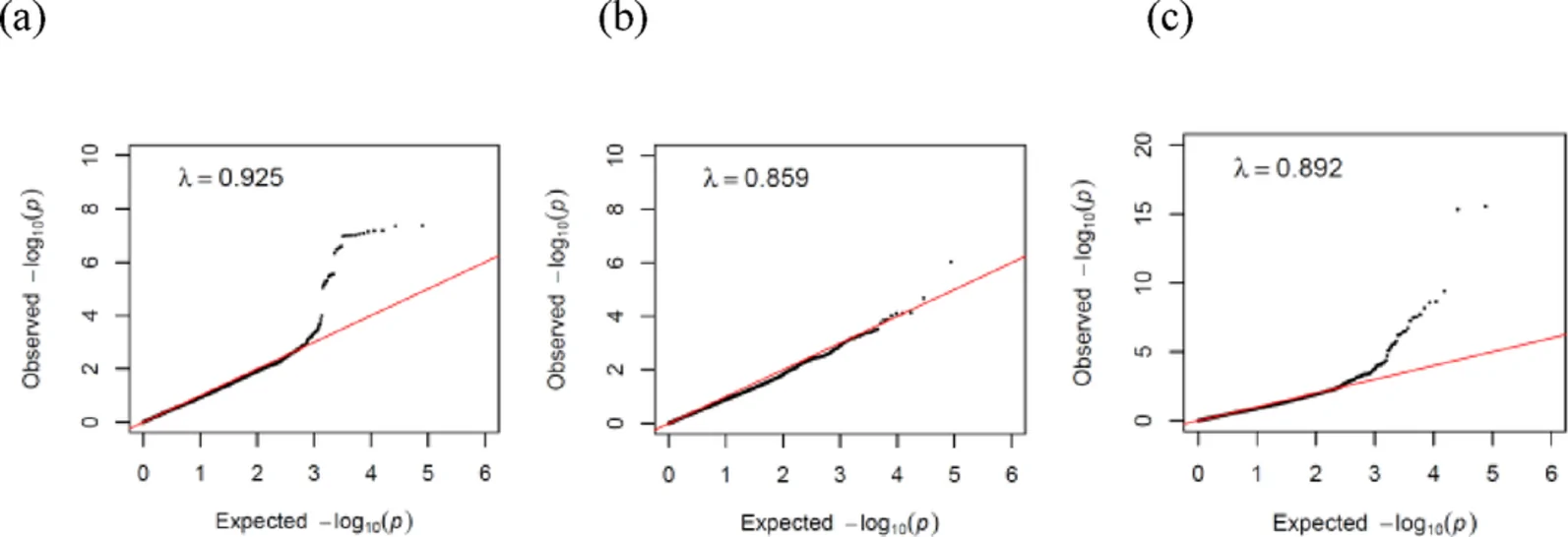
Quantile–quantile plots of the genome-wide association studies for genetic effect of sow on Number Born Alive for **a** Line A **b** Line B **c** Line C

Manhattan plots for the genetic effect of sow on NBA for the three lines are shown in Fig. 3. Significant peaks were identified for all three lines. For line A, 18 significant SNPs were located on chromosome 6 151.23 Mb, with -log10(P-value) ranging from 6.3 to 7.4. For line B, one significant SNP was identified on chromosome 1 near 192.08 Mb, with a -log10(P-value) of 6.0. For line C, 16 significant SNPs were detected, with -log10(P-values) ranging from 6.2 to 15.6. These SNPs were located on chromosome 1 (at 14.34 to 14.45 Mb), chromosome 2 (at 150.48 Mb), chromosome 3 (at 93.80 to 94.37 Mb), chromosome 5 (at 83.05 Mb), and chromosome 9 (at 117.86 to 137.15 Mb). Quantile–quantile plots of the GWAS for the genetic effect of sow on NBA are in Fig. 4. The λ of the QQ plot regressions were 0.93, 0.86, and 0.89 for lines A, B, and C, respectively, indicating slight deflation for all three lines.

## Discussion

### Service sire contribution to litter size variation

Over the past few decades, several studies have investigated the influence of paternal components, both genetic and non-genetic, on sow reproductive performance. In earlier research, See et al. [1] reported that, although the service sire effect was small, it was significant, accounting for 1 to 2% of the total variation in number born alive in Landrace, Hampshire, and Spotted populations. More recently, Cieleń and Sell-Kubiak [3] reviewed studies assessing the proportion of phenotypic variance in reproductive traits explained by the service sire and found that the genetic component accounted for 1 to 2%. The non-genetic service sire effect was slightly higher, ranging from 1 to 5.5%, though this may partly reflect confounding with genetic effects in models where the service sire genetic component was not included. The low heritability estimates for the service sire effect on NBA reported in our study fall within the range of previously published values.

### Modeling service sire effects

We modeled the service sire and sow genetic effects with additive relationship and covariances between them when estimating the genetic parameters. Although this modeling approach has been adopted in previous studies for genetic parameter estimation in pigs [8–10], it remains relatively uncommon, possibly due to software limitations or the complexity of the modeling process. Furthermore, we extended this approach to GEBV estimation and GWAS, whereas previous studies did not. Accounting for the additive relationship and covariances between the service sire and sow genetic effects, Serenius et al. [8] investigated the effect of service sire on litter size in Finnish Landrace and Large White populations. They reported near zero estimates of variance due to service sire on litter size, whereas the heritability of sow effect ranged between 0.11 and 0.15. Van der Lende et al. [9] analyzed one purebred sire line, two purebred maternal lines and one F1 line from the Netherlands and considered the additive relationship and covariances between the service sire and sow genetic effects. For purebred genetic parameter estimates, they reported heritability estimates for the service sire effect on litter size ranging from 0 to 0.04, whereas sow heritability estimates were nearly threefold higher, ranging from 0.07 to 0.12. Kim et al. [10] also considered the additive relationship and covariances between the service sire and sow genetic effects and reported sow and service sire genetic parameters for litter size and birth weights in a South Korea Yorkshire population. Estimates of heritability of sow effect were lower for litter size (0.18 to 0.19) than for birth weight traits (0.25 to 0.39). Service sire heritability estimates ranged from 0.01 to 0.02 for all traits.

Having service sire as a non-genetic random effect without considering the additive relationship among sires or between sires and sows, Wolf and Wolfová [35] reported sow heritability estimates of 0.05 to 0.07 for number of stillborn and number of piglets lost from birth until weaning in Czech Landrace and Czech Large White population. The percentage of variance due to service sire was very low, between 1 and 1.2% for number of stillborn and between 0.8 and 1.6% for piglets loss. Chen et al. [4] studied number born alive and weaning performance in U.S. Landrace, Yorkshire, Duroc, and Hampshire populations. For number born alive, heritability estimates for the sow effect ranged from 0.08 to 0.10, while the percentage of variance due to service sire effects ranged from 2 to 4%. Serenius et al. [5] reported that the percentage of variance due to the service sire effect ranged from 1 to 2% for litter size traits, from 1 to 4% for piglet survival traits, and noticeably higher, from 5 to 9% for gestation interval. Cielen´ et al. [7] investigated litter size variability in Landrace using a model that included both genetic and non-genetic service sire effects, while assuming no additive relationships between service sire and sow. They also reported the genetic variance attributable to the service sire to account for less than 2% of the phenotypic variance for litter size.

Our estimates of the genetic correlation (SE) between the service sire and sow effects were − 0.20 (0.26), 0.37 (0.22), and 0.23 (0.11) for lines A, B, and C, respectively. The estimate was negative for line A but with a relatively large standard error as the data set for this line was smaller than for lines B and C. This negative estimate could, therefore, be the result of sampling variation. Van der Lende et al. [9] reported estimates of the genetic correlation between service sire and sow effects for the three lines were 0.08, -0.37, -0.47 for total number born and 0.10, 0.20, and − 0.20 for number born alive. Standard errors for the estimates were not provided. Kim et al. [10] reported estimates of the genetic correlation between service sire and sow effects were 0.01 to 0.11 for litter size and ranged from − 0.15 to 0.54 for birth weights traits. No SE were provided.

Our estimates of the permanent environmental variance for the service sire effect was less than 1% of the phenotypic variance for NBA. Several studies omitted permanent environmental effects of the service sire from the models [4, 9, 10, 35]. Van der Lende et al. [9] omitted the service sire permanent environmental effect after preliminary analyses indicated a very small variance component. Wolf and Wolfová [35] excluded the service sire permanent environmental effect and instead modeled the service sire as an independent and identically distributed random term, without separating its genetic and permanent environmental components. Whether treating the service sire as an independent random effect or as an additive genetic effect with or without a permanent environmental component, literature results reflect a consistent pattern that the variance attributable to the service sire is much smaller than that of the sow. Because the service sire contributes only a minor portion of the total variation in litter size, estimating its permanent environmental component in addition to the genetic component is inherently difficult and prone to convergence issues, especially when the data set is small, as it did for line A. In contrast, the model that included permanent environmental effects for the sow but not the boar converged successfully for this line.

### Genetic parameters of semen quality traits

Our heritability estimates for semen motility (0.10 to 0.12) and abnormal morphology (0.17 to 0.28) fall within the range of values reported in the literature [6, 13, 14, 36]. Morphology can be analyzed based on individual traits or as a composite measure. The genetic parameter estimates summarized herein focus on morphology defined as a total score.

Gruhot et al. [12] analyzed semen quality traits on ejaculates collected during the summer season in the United States on about 400 Duroc boars and found heritability estimates ranging from 0.08 to 0.24. Ogawa et al. [13] analyzed semen traits 46,000 ejaculates from 900 Duroc boars and estimated heritability of about 0.20 with repeatability of 0.46 for proportion of morphologically normal sperm. For Landrace and Large White, Wolf [6] estimated heritability in Czech Landrace and Large White pigs of around 0.1 for motility and morphology based on 37,000 to 51,000 ejaculates from about 800 boars per breed. Marques et al. [36] reported average heritability of 0.18 for motility and 0.20 for morphology across five pig lines (Duroc, Pietrain, Landrace, Large White, and a synthetic line). Krupa et al. [37] analyzed 57,000 sperm collections records from 1209 Czech Landrace and Large White boars and reported heritability estimated of 0.11 to 0.14 for motility and 0.22 to 0.24 for morphology. Sa et al. [14] analyzed semen traits collected from 450,000 ejaculates on about 5700 boars from a commercial synthetic line. For motility, estimates of heritability ranged from 0.20 to 0.24, with repeatability ranging from 0.35 to 0.58. For total morphological abnormalities, the estimate of heritability was 0.21 with a repeatability of 0.52. For other sperm morphology traits, heritability ranged from 0.11 to 0.27, with repeatability ranging from 0.20 to 0.65. Overall, the reported heritability estimates from literature and this study for morphology were similar or higher than estimates for motility.

The three lines analyzed here represent independent populations with different breed origins and breeding objectives. Consequently, differences in variance estimates across lines are expected. Such differences may arise from long-term evolutionary processes, genetic drift, distinct selection histories, and differences in underlying biological variation between lines. These factors can collectively influence the amount of genetic and non-genetic variation expressed within each line. In addition, the data sets used in this study were derived only from farms that had implemented CASA technology. Consequently, the analyzed animals represent a subset of the overall population rather than all commercial farms, and potential sampling variation may have contributed to the observed differences in the estimates.

### Relationships among traits

The negative genetic correlations between motility and morphology were observed in the current study with a range from − 0.64 to -0.34. The negative genetic correlations were also reported in literature [6, 12, 36, 37]. For example, the genetic correlation of -0.66 was reported in Marques et al. [36] and estimates between − 0.42 and − 0.35 were reported in Krupa et al. [37].

For genetic correlations between litter size and semen traits, Wolf [6] reported that those genetic correlation estimates could be breed-specific, especially between litter size and the percentage of abnormal sperm. Ogawa et al. [13] estimated the genetic correlation between normal morphology and litter size to be low, 0.12, 0.18 and − 0.17 for total number born, born alive, and stillborn, and with high SE but in a favorable direction. Our GEBV correlations between litter size and semen quality were low but favorable, and some had slightly large standard errors. For semen morphology, our GEBV correlations were similar or lower in magnitude than the genetic correlations reported by Ogawa et al. [13]. Although the sign of the correlation differed due to opposite trait definitions (abnormal vs. normal morphology), the direction of the biological association with litter size was in agreement. Overall motility and abnormal morphology had, respectively, positive and negative GEBV correlations with litter size, suggesting that selection on semen quality traits can increase litter size. Although our low GEBV correlations between litter size and semen quality may be partly attributed to the small number of service sires used in the correlation analyses, they also suggest that paternal contributions to litter size have not been fully accounted for. Incorporating paternal contributions on litter size, alongside semen quality, into selection decision could therefore enhance the overall assessment of boar reproductive performance.

### Genome-wide association analysis of service sire effects

To our knowledge, this study was the first GWAS analysis on service sire genetic effects for litter size in pigs. We identified only one significant marker associated with the service sire effect for NBA, in line B, with a -log10(P-value) that was only slightly above the significance threshold. While more significant markers were identified for sow genetic effects on NBA, the objective of our GWAS analysis was to focus on the service sire effect.

Although the additive genetic variance of the service sire effect was low for the three lines (0.28, 0.08, and 0.15, with SEs of 0.08, 0.03, and 0.04, respectively), as were the corresponding heritability estimates (0.03, 0.01, and 0.02 with SEs of 0.008, 0.004, and 0.005), the associated standard errors indicate that the service sire effect on NBA was significantly different from zero. Thus, the absence of significant results from the GWAS for the service sire genetic effect may reflect the highly polygenic architecture of NBA, in which many loci contribute small individual effects. Moreover, because service sire effects accounted for a smaller proportion of the additive genetic variance than sow effects, the genetic variants underlying the service sire genetic effect are likely to have relatively small individual effects, making them more difficult to detect. For highly polygenic traits with very low heritability, the resulting low reliability of GEBVs may also influence the precision of marker effects estimates, which in return may affect the detection of statistically significant GWAS associations. Jang et al. [38] conducted a simulation study and showed that GWAS detected more true positive causal variants when the genotyped individuals had high-reliability GEBVs.

Leite et al. [23] showed the same significance peaks and similar p-values when comparing ssGWAS with and without APY using a large beef cattle dataset. The APY-based ssGWAS approach makes it computationally feasible to obtain p-values in large genotyped populations, providing an additional interpretable measure of statistical significance in addition to SNP effect estimates and variance explained. Increasing the number of genotyped animals under this framework is particularly beneficial for strengthening the power to detect associations, especially for sex-limited traits, and helps avoid bias arising from analyses restricted to subsets of genotyped animals.

## Conclusions

Based on large data sets of 1 million ejaculates from 50,000 boars from three terminal sire lines, this study reports low to moderate heritability and moderate to high repeatability estimates for semen motility and total abnormal morphology traits. Moderate and favorable genetic correlation estimates between semen quality traits indicate that selection for semen motility may be favorable for improving morphology. The genetic impact of the service sire effects on litter size appeared to be small, as indicated by the low heritability estimates and the limited number of significant markers detected. Although paternal genetic contributions to litter size were small compared to maternal genetic contributions, selecting for service sire effects on litter size in addition to semen quality traits is expected to improve boar reproductive performance.

## Data Availability

The dataset used in this study was obtained from a pre-existing dataset owned by Genus PIC and not publicly available.
